# Advance Care Planning on a Specialist Dementia Unit

**DOI:** 10.1192/bjo.2025.10345

**Published:** 2025-06-20

**Authors:** Jo Butler, Asli Ozcivan, Tharun Zacharia

**Affiliations:** South London and the Maudsley NHS Foundation Trust, London, United Kingdom

## Abstract

**Aims:** Advance care planning is a process of person-centred discussion between individuals and their care providers about future care preferences. Advance care planning discussions are relevant for those wishing to plan for their future care or those at increased risk of losing mental capacity, such as dementia patients or individuals with life-limiting illnesses. Aims were:

Improve advance care planning discussions on a specialist dementia unit.

Improve identifying patients who would benefit from an Advance Care Plan (ACP).

Offer all patients an initial ACP discussion.

Ensure patients with an estimated prognosis of <12 months have an ACP in place by discharge.

Improve ACP communication to acute hospitals and GPs.

**Methods:** Patient data was collected weekly from May 2024 until January 2025. 67 patients in total.

The Universal Care Plan (UCP) was chosen as a framework to document ACPs. The UCP is an NHS service that enables patients in London to have their care and support wishes digitally shared with healthcare professionals across the capital.

The Gold Standards Framework Proactive Identification Guidance was used to identify patients at risk of physical deterioration and prioritisation for ACPs. Prognostic coding and prioritisation for ACPs was reviewed daily with the MDT.

Staff underwent training on ACPs. More accessible ACP information was provided on the unit. The process of booking ACP discussions was refined, introducing a weekly ACP meeting slot (starting 23/07/24).

**Results:** Number of patients on the unit with an ACP increased between May 2024 and January 2025, from a low of 0% on 03/06/24 to a high of 53% on 12/12/24. Initial ACP discussions offered increased from 27% (20/05/24) to 93% (28/01/25). After introducing the weekly ACP slot, 41% slots were filled. 48% of dementia patients had an ACP by discharge. 50% patients with an estimated <12-month prognosis had an ACP by discharge.

By January 2025, 100% prognostic coding was communicated to the GP.

**Conclusion:** The weekly timeslot increased the number of ACPs. Improved identification of patients resulted in approximately half of dementia patients, and half of patients with a prognosis of months, having an ACP by discharge. Challenges included embedding ACP meetings into routine practice. Recommendations:

Gather feedback from patients and carers – case studies suggested ACP discussions were well received by carers and positively impacted patient care.

Continue the regular weekly ACP slot.

Provide more staff training.

Audit outputs of ACP discussions.

